# 4492 The role of creatine in developmental myelination and remyelination

**DOI:** 10.1017/cts.2020.318

**Published:** 2020-07-29

**Authors:** Lauren Rosko, Victoria N Smith, Jeffrey K. Huang

**Affiliations:** 1 Georgetown - Howard Universities; 2 Georgetown University

## Abstract

OBJECTIVES/GOALS: Oligodendrocytes (OL) are glial cells of the central nervous system (CNS) responsible for the energy demanding task of generating myelin sheaths during development and remyelination after demyelinating injury. One metabolite shown to significantly increase ATP production in OL is the nitrogenous organic acid, creatine. Creatine plays an essential role in ATP buffering within tissues with highly fluctuating energy demands such as brain and muscle. Interestingly, mature OL, which are the cells capable of myelin production, are the main cells in the CNS expressing the rate-limiting enzyme for creatine synthesis, guanidinoacetate methyltransferase (*Gamt)*. Patients with mutations in *Gamt* display intellectual disabilities, impaired myelination and seizures. Therefore, we hypothesize that creatine may be essential for developmental myelination and improve remyelination. METHODS/STUDY POPULATION: To investigate these hypotheses, we developed a new transgenic mouse model with LoxP sites flanking exons 2-6 of the *Gamt* gene where excision leads to expression of a green fluorescent tag allowing us to track the cells normally expressing *Gamt.* RESULTS/ANTICIPATED RESULTS: In this mouse model, we show a 95% (±0.47%, n = 3) co-localization of *Gamt* within mature OL during postnatal (P) day P14. Next, we show that knocking out *Gamt* leads to a significant reduction in OL in the major CNS white matter tract, the corpus callosum, at P14 and P21 (P14: 0.007, n = 3; P21: 0.04, n = 3). Here, we also investigate whether dietary creatine can enhance remyelination in the cuprizone model of toxic demyelination. DISCUSSION/SIGNIFICANCE OF IMPACT: These studies highlight the important role creatine plays in developmental myelination and investigate whether creatine can provide a therapeutic value during a CNS demyelinating insult.

